# Bank loan information and information asymmetry in the stock market: evidence from China

**DOI:** 10.1186/s40854-022-00367-0

**Published:** 2022-05-24

**Authors:** Yanyi Ye, Yun Wang, Xiaoguang Yang

**Affiliations:** 1 School of Economics and Management, Beijing University of Chemical Technology, Beijing, 100029 China; 2 School of Banking and Finance, University of International Business and Economics, Beijing, 100029 China; 3 Academy of Mathematics and Systems Science, Chinese Academy of Sciences, Beijing, 100190 China; 4 University of Chinese Academy of Sciences, Beijing, 100049 China

**Keywords:** Bank loan information, Information asymmetry, Corporate transparency, Loan default information, PIN, G12, G14, G21

## Abstract

In this study, we use bank loan information to construct proxies for corporate transparency and examine whether these measures reflect information asymmetry in the stock market. Our analysis is based on a novel dataset of stock transactions and bank loans of all publicly listed firms on the Shenzhen Stock Exchange, covering January 2008 to June 2013. We find that firms with outstanding loans have a lower level of information asymmetry in the stock market, whereas firms with defaulted loans have a higher level of asymmetry. Further evidence demonstrates that the effect of loan default on information asymmetry in the stock market is more pronounced when these loans are borrowed from joint-equity commercial banks or multiple banks and when the default occurs under inactive market conditions. Our results remain robust to a series of endogeneity and sensitivity tests and provide suggestive evidence of a close connection between the credit loan and stock markets.

## Introduction

Information asymmetry between informed and uninformed investors is fundamental in the security market. Since information asymmetry in the stock market varies with the cross-sectional variation in corporate transparency, the construction of reliable measures for corporate transparency has long been a concern for researchers (Bushman et al. 2004; Miller 2004; Leuz and Oberholzer-Gee 2006; Andrade et al. 2014; Balakrishnan et al. 2019). However, corporate transparency cannot be observed directly, and traditional measures mainly focus on corporate disclosure and firm fundamentals (Roulstone 2003; Bushman et al. 2004; Miller 2004; Sankaraguruswamy et al. 2013; Firth et al. 2015; Glaeser 2018). This study explores the relationship between bank loan information and stock market information asymmetry. Specifically, we use bank loan information to construct proxies for corporate transparency and investigate whether these measures reflect information in the stock market.

Traditional proxies for corporate transparency are mainly based on corporate disclosures, such as financial statements, management meetings, and regulatory filings, which is one of the most important means through which managements share information about their firm’s performance and governance with investors (Healy and Palepu 2001). Generally, corporate disclosure generates cost savings for investors attempting to acquire valuable information, and increased disclosure can reduce information asymmetry and decrease a firm’s cost of capital (Diamond 1985; Diamond and Verrecchia 1991). Bushman et al. (2004) argue that corporate transparency has three dimensions: corporate reporting, information dissemination, and private information acquisition. However, proxies for corporate transparency constructed from corporate disclosures mainly focus on the first two dimensions. Moreover, firms have substantial discretion in corporate disclosure, often engaging in reporting good news and minimizing or withholding bad news because of managers’ career and compensation concerns (Hossain et al. 1995; Lang and Lundholm 1996; Hutton et al. 2009; Jin and Myers 2006). This voluntary nature of corporate disclosure results in the public becoming better informed about a company’s good news than bad news. Therefore, traditional proxies for corporate transparency constructed from corporate disclosures are likely to be biased.

Compared with corporate disclosure, bank loan information has several unique features that correlate with some aspects of corporate transparency. On the one hand, banks have access to borrowers’ private information and are treated as insiders (Bushman et al. 2010; Ivashina and Sun 2011; Ma et al. 2019). The origin and ongoing maintenance of a bank loan depends on the private material that borrowers provide (Dennis and Mullineaux 2000; Sufi 2007). However, such privileged information is not always publicly available. On the other hand, the ongoing lending relationship motivates banks to monitor their borrowers closely and increases disclosure, even for negative information (Bushman et al. 2004; Acharya and Johnson 2007; Ivashina 2009). These features make it possible to construct proxies for corporate transparency using loan information that captures more private and negative information than traditional information.

Meanwhile, loan information catches different levels of information asymmetry. First, firms with outstanding loans present an avenue for higher corporate transparency. Borrowers must disclose their proprietary information to the bank to obtain bank loans, such as timely financial statements, covenant compliance information, amendments and waiver requests, financial projections, and acquisition plans (Standard 2010). Moreover, banks’ screening and monitoring activities increase information dissemination, which provides incentives to hide negative information; however, borrowers must report their information to lenders in a timely and accurate manner (Ma et al. 2019; Gustafson et al. 2021; Bhat and Desai 2020). Further, the existence of a bank loan also reduces information asymmetry between firms and outside investors through loan announcements (Maskara and Mullineaux 2011a).

Second, firms with defaulted loans have lower levels of corporate transparency. Banks tend to act as efficient monitors to reduce the default probability of loans (Dennis and Mullineaux 2000; Sufi 2007; Bushman et al. 2010). When the loan defaults, the bank cannot monitor the ambiguous changes in the firm efficiently because financially distressed firms tend to hide negative information from lenders and update their private information to lenders with low frequency; this leads to the failure to repay a loan and a lower level of corporate transparency (Sufi 2007; Kim 2020).

This study examines the relationship between bank loan information and information asymmetry in the stock market. Our analysis is based on a novel dataset of stock transactions and loan information for all publicly listed firms on the Shenzhen Stock Exchange (SSE) from January 2008 to June 2013. The loan-level data are from the regulatory body, and we construct measures for corporate transparency based on this loan information.^1^ Specifically, we identify a higher level of corporate transparency for firms with outstanding loans and a lower level of corporate transparency for firms with defaulted loans. To identify loan default, we trace the events of a loan becoming overdue and being signed as a *NPL* (non-performing loan). For stock market analysis, we employ the EHOH model developed by Easley et al. (2002) to estimate the probability of information-based trading (PIN) as the baseline measure of information asymmetry in the stock market.

Our main empirical strategy examines whether proxies of corporate transparency constructed from loan information reflect information asymmetry in the stock market. Our main findings are as follows: First, firms with outstanding loans exhibit a decrease of 28 basis points in *PIN*, which is our baseline measure of information asymmetry in the stock market. Moreover, a one-standard deviation (1532 million) increase in loan size leads to a 35.24 basis points reduction in *PIN*. The reduction in *PIN* is significantly larger when loans are borrowed from a joint-equity commercial bank. These findings are consistent with our expectations and imply a relationship between positive loan information and information asymmetry in the stock market.

Second, we examine whether informationally opaque firms (those with a lower level of corporate transparency), as measured by loan defaults, have a higher level of information asymmetry in the stock market. Our results demonstrate that among firms with overdue loans, *PIN* increases by 1.16, and a one-standard-deviation increase in the overdue loan rate is related to a 39 basis points increment in *PIN*. We also find that the effect of negative loan information on information asymmetry in the stock market is more pronounced if these loans are borrowed from joint-equity commercial banks or multiple banks. Moreover, our results demonstrate that the marginal effect of negative loan information’s impact on information asymmetry in the stock market is much larger than that of positive loan information; this suggests that bank loan information can effectively supplement corporate disclosure because the latter is biased toward positive information.

Third, we further examine the effect of both positive and negative loan information on information asymmetry in the stock market under different market conditions and find that these effects decrease under active market conditions.

Finally, our results are robust to a series of endogeneity and robustness tests. First, firms may have specific characteristics related to obtaining loans and determining corporate transparency simultaneously, which may lead to sample selection bias. To eliminate these possible selection biases, we include firm-fixed effects in all regressions to control for time-invariant heterogeneity and conduct an analysis using the Heckman two-stage selection model. Second, to demonstrate that loan information captures different aspects of corporate transparency compared to corporate disclosure, we add analyst coverage (*Analyst*) as a control variable in all regressions. Third, bank loan information can be shared with stock markets through means other than public disclosure, such as site visits. However, some funds and securities firms belong to the same business groups as banks or are directly held by banks. Further, informed banks may share information with related financial institutions. In a subsample analysis, we remove the observations that contain publicly announced loan information, and our findings remain the same.^2^ Fourth, to eliminate the concern regarding measurement errors, we use *VPIN* (Easley et al. 2012) and *Bid-Ask Spread* (Lee et al. 1993; Madhavan et al. 1997) to replace *PIN* as a measure of information asymmetry in the stock market and use new loan information and non-performing loans to replace outstanding loans and overdue loans. The results based on these alternative measures are consistent with those of the baseline models.

Our main contributions are threefold. First, we contribute to the literature on how to measure corporate transparency (Bushman et al. 2004; Miller 2004; Leuz and Oberholzer-Gee 2006; Andrade et al. 2014; Balakrishnan et al. 2019). We construct proxies for corporate transparency using loan information and demonstrate that they capture aspects of corporate transparency other than disclosure. Second, our results reveal a relationship between loan information and information asymmetry in the stock market. Previous studies have focused on how bank monitoring can reduce information asymmetry between borrowers and lenders (Bhat and Desai 2020; Datta et al. 1999; Gustafson et al. 2021; Ma et al. 2019; Booth 1992). Our results indicate that firms with outstanding loans have lower levels of information asymmetry in the stock market. Our findings are consistent with a series of studies demonstrating that the stock market reacts to information in the credit market, such as private information in the loan market accelerating price discovery in the stock market (Bushman et al. 2010), institutional investors trading on loan market information (Ivashina and Sun 2011), and short selling in the stock market through hedge funds accessing private information in the syndicated loan market (Massoud et al. 2011). Finally, a growing body of the literature focuses on the effect of financial distress on information asymmetry in the stock market (Franks and Sussman 2005; Piri et al. 2020; Salehi et al. 2020). In line with this body of literature, our results demonstrate that loan defaults are a good proxy for a lower level of corporate transparency and that informationally opaque borrowers will have a higher level of information asymmetry in the stock market.

Overall, our empirical analyses shed light on the important role of proxies for corporate transparency constructed from loan information. Moreover, our results suggest that positive (negative) loan information can reflect a lower (higher) level of information asymmetry in the stock market.^3^

The remainder of this paper is organized as follows. “Literature review and hypotheses development” section addresses the literature and proposes the hypotheses. “Research design and data” section describes the research design and summarizes the data, methods, and explanatory variables. “Empirical results” section presents the empirical results, and “Additional analyses” section reports the additional analyses and robustness checks. Finally, “Conclusion” section concludes the study.

## Literature review and hypotheses development

### Theoretical discussion

*Information asymmetry in the lending market* Compared with other financial intermediaries, banks are treated as insiders; this is because they can collect proprietary information about firms through the screening and monitoring process, which mitigates the information asymmetry between the transaction parties (Petersen and Rajan 1994; Berger and Udell 1995; Maskara and Mullineaux 2011a). Simultaneously, because of the use of covenants and collateral, banks can act as efficient monitors because collateral can mitigate adverse selection and moral hazards (Ioannidou et al. 2022; Rajan and Winton 1995). According to the models developed by Holmstrom (1979) and Holmstrom and Tirole (1997), firms with limited public information require monitoring by an informed lender before uninformed lenders invest in them. However, there is an adverse selection problem because banks have an incentive to syndicate risky loans. Information sharing among lenders can mitigate this adverse selection problem (Pagano and Jappelli 1993). In addition, a moral hazard problem exists for the informed lender because the informed lender’s monitoring is unobservable. To solve this problem, an informed lender with monitoring and due diligence responsibilities must retain a large share of loans (Sufi 2007; Ivashina 2009).

*The informational role of debt* According to the seminal work of Harris and Raviv (1990), the informational role of debt comes from two aspects. First, a firm’s ability to make interest and principal payments provides information about its operation and fundamentals. Second, defaults can disseminate considerable information to investors because debtholders can use their legal rights to force managements to provide information and make efficient liquidation decisions. The model developed by Habib and Bruce (2015) emphasizes that debt and equity reveal different aspects of information as equity reveals information about the enterprise in its primary use while debt reveals information about the enterprise in its alternative use. Many empirical studies support the theory of the information role of debt. In a recent study, Ghorbani and Salehi (2020) conduct an analysis based on data from Iran and found that the use of higher leverage contributes to a reduction in agency costs, which is consistent with the theory.

*Information asymmetry in the stock market* The information asymmetry between informed traders and uninformed traders in the stock market is one of the fundamental issues in the market microstructure. The price effect of asymmetric information has been analyzed in a series of studies, such as Kyle (1985) and Glosten and Milgrom (1985). Meanwhile, based on the market microstructure model derived by Easley et al. (2002), information differences across investors generate information asymmetry. Informed traders hold private information, buy if they have received good news, and sell if they have received bad news. Less informed traders recognize that they are at an information disadvantage and hold fewer assets (Easley et al. 1996, 1997a, b, 2002; Lambert et al. 2011). At equilibrium, uninformed traders require compensation to hold stocks with greater private information. Duarte and Young (2009) extend the EHOH model that allows for the possibility of symmetric order-flow shocks. All theories emphasize the different reactions of informed traders to good private news and bad private news.

According to the theories mentioned above, bank loan information is related to the information asymmetry of borrowers in the credit market and can reveal different aspects of information compared to equity. Therefore, informed traders may take advantage of this information to conduct informed trading in the stock market. This study provides insights into these theories to examine the effect of loan information on information asymmetry in the stock market.

### Literature review and hypotheses development

There has been a noticeable increase in the literature on the nature of the relationship between corporate disclosure and information asymmetry. Healy and Palepu (2001) point out that transparency in financial reporting reduces information asymmetry between corporate managers and capital suppliers. Diamond and Verrecchia (1991) demonstrate that corporate disclosure can lower a firm’s cost of capital by reducing information asymmetry in the securities market and by improving the firm’s future liquidity. Shroff et al. (2013) also find that voluntary disclosure is related to a decrease in information asymmetry and a reduction in the cost of raising equity capital. Nagar et al. (2019) find that managers may supply additional voluntary disclosure if information asymmetry among investors increases. Cheynel and Levine (2019) demonstrate that the informed trader’s ability to combine information and enhance their advantage is more prevalent when there is more uncertainty about whether the news is favorable or unfavorable. Despite a rich body of literature focusing on information asymmetry (e.g., Asongu et al. 2019; Pan and Misra 2020; Ha et al. 2021; Zhao 2021; Ioannidou et al. 2022; Cheynel and Levine 2019; Nagar et al. 2019), few studies investigate the relationship between proxies for corporate transparency constructed from loan information and information asymmetry in the stock market.

Bank loan information has become a more noticeable concern in the literature, and existing studies reveal that bank loan information decreases the information asymmetry between lenders and borrowers. Maskara and Mullineaux (2011a) demonstrate that bank loan announcements are relatively rare events that are commonly driven by information asymmetry and perceived materiality. Ivashina (2009) investigates how the availability of information about borrowers directly affects the information asymmetry between the lead bank and the syndicate participants. Acharya and Johnson (2007) find that, because of bank monitoring, the degree of information flow increases as the number of banks with ongoing lending relationships with a given firm increases. Ioannidou et al. (2022) provide evidence that collateral mitigates asymmetric information in lending markets. Moreover, information asymmetry in banking in emerging markets has been discussed in the recent literature (Tsindeliani and Mikheeva 2021; Ghorbani and Salehi 2020; Faysal et al. 2021).

Bank loans provide effective corporate disclosure in the equity market. Bushman et al. (2010) document that borrowers disseminating private information to lenders indeed exhibit faster price discovery in the stock market. Ivashina and Sun (2011) find that institutional investors have access to the private information disclosed during loan amendments, trade in the stock market, and obtain more abnormal returns compared to other investors who do not have that access. Massoud et al. (2011) also find evidence that the equity of hedge fund borrowers is short-sold before public announcements regarding loan origination and loan amendments.

Bank loan information will increase corporate transparency, as lenders and loan providers prefer access to borrowers’ private information to stay informed and ensure the timely repayment of loans and interest (Ma et al. 2019). Lenders monitor borrowers on the loan market (Bhat and Desai 2020), and financial covenants often obligate borrowers to provide timely covenant reports and private information to lenders, which further reduces borrower–lender information asymmetry (Bhattacharya and Chiesa 1995; Bushman et al. 2010). For example, half of the lenders in one study required borrowers to provide information at least monthly (Gustafson et al. 2021). On its own, a loan can lower the information asymmetry between a borrowing firm and its investors (Petersen and Rajan 1994; Berger and Udell 1995; Maskara and Mullineaux 2011a), and the larger the loan amount, the stronger the incentive for banks to monitor (Ma et al. 2019). Based on these prior findings, we believe that firms exposed to lending have better corporate transparency, as can be observed in the stock market. Moreover, loan lending from a joint equity commercial bank provides greater corporate transparency. Compared with state-owned banks, joint-equity commercial banks are not responsible for issuing loans to politically connected firms unrelated to their creditworthiness. Thus, loans issued by joint-equity commercial banks are more market-oriented and contain more information about borrowers’ creditworthiness. Therefore, we predict that bank loans will lower the level of information asymmetry in the stock market, as follows:

Hypothesis 1 (H1). Firms with outstanding bank loans have lower information asymmetry in the stock markets.

H1a: The level of information asymmetry in the stock market decreases when a firm has outstanding loans.

H1b: The level of information asymmetry in the stock market decreases relative to an increase in loan amounts.

H1c: The level of information asymmetry in the stock market decreases when loans are borrowed from joint-equity commercial banks.

Banks frequently demand information on borrowers, and there is substantial cross-sectional heterogeneity in this demand for information (Gustafson et al. 2021). Different borrowers are required to report to lenders at different periods. Financially distressed firms tend to hide negative information from lenders and update private information to lenders with low frequency, and they are more likely to fail to repay loans.

As financially distressed firms tend to hide bad news, information asymmetry in the stock market increases. Borrowers tend to hide bad news and are more likely to announce loans when they demonstrate considerable information asymmetry to investors or when they make up a large component of the borrower’s capital structure (Maskara and Mullineaux 2011a). Banks increase monitoring following deteriorations in borrowers’ financial condition and credit line drawdowns (Kim 2020). However, the existence of a lending relationship with a distressed firm still results in wealth declines for banks (Franks and Sussman 2005; Piri et al. 2020; Salehi et al. 2020). Ivashina and Sun (2011) point out that bad news regarding loan amendments leads to negative stock returns. Consequently, we predict that negative loan information leads to a higher level of information asymmetry in the stock market.

Acharya and Johnson (2007) propose that information flow across markets occurs only for negative credit news, and this information flow increases when the number of lending banks increases. However, even when monitored by multiple banks, financially distressed firms still default, indicating that financially distressed firms are information opaque. Meanwhile, loans issued by joint-equity commercial banks contain more information about borrowers’ creditworthiness. Therefore, we predict that the asymmetric information effects^4^ of loan defaults will be more pronounced when loans are borrowed from a joint-equity commercial bank or multiple banks.

Hypothesis 2 (H2). Financially distressed firms with defaulted loans have a higher level of information asymmetry in the stock market.

H2a: The level of information asymmetry in the stock market increases when a firm has loans that they fail to pay.

H2b: The level of information asymmetry in the stock market increases with the default loan ratio.

H2c: The asymmetric information effects of loan defaults are particularly pronounced when loans are borrowed from a joint-equity commercial bank.

H2d: The asymmetric information effect of loan defaults is particularly pronounced when the number of lending banks increases.

The asymmetric information effects of loan information vary with variability in the activity of market conditions. Under active market conditions, there is a growing amount of information in the security market, and this information captures investors’ attention. As investors face limited attention constraints, they may not be able to pay additional attention to loan information. Therefore, loan information has fewer effects on the stock markets. However, information obtained from the loan market will be more valuable under inactive market conditions because of the lack of public disclosure. Bushman et al. (2010) find that the positive relationship between institutional lending and the speed of stock price discovery is more pronounced in relatively weak public disclosure environments. Subsequently, we predict that the asymmetric information effects will be lower under active market conditions.

Hypothesis 3 (H3).

H3a: The negative relationship between corporate transparency and information asymmetry in the stock market is less pronounced under active market conditions than under inactive ones.

H3b: The asymmetric information effects of loan defaults are less pronounced under active market conditions than under inactive ones.

## Research design and data

### The measure of information asymmetry in the stock market

*PIN* (the probability of information-based trading) is a good proxy for information asymmetry in the stock market described in Easley et al. (2002). We use the EHOH structural model to measure information-based trading, derived from a series of microstructure studies (Easley et al. 1996, 1997a, b, 2002).

The EHOH model is used to construct the theoretical opening bid and ask prices based on the estimated model parameters. This model indicates that a market maker sets trading prices based on their expected losses to informed traders to offset the expected gains from trading with uninformed traders in standard microstructure models. The balancing of gains and losses is spread between bid and ask prices, thereby allowing the interpretation of information-based trading. Opening trade, *PIN*, is computed as follows:

1 $$\begin{aligned} PIN=\frac{\alpha \mu }{\alpha \mu +\epsilon {_{s}}+\epsilon {_{b}} } \end{aligned}$$

where $\alpha \mu +\epsilon _{s}+\epsilon _{b}$ is the arrival rate of all trades and $\alpha \mu$ is the arrival rate of information-based trades. Therefore, *PIN* is the ratio of the arrival rate of information-based trades to the arrival rate of all trades, which is the fraction of orders that emerge from informed traders or the probability of the opening trade being information-based. Recent studies that use *PIN* as a measure of the probability of informed trading (Bennett et al. 2020; De Angelis et al. 2017; Friewald and Nagler 2019; Manconi et al. 2018) find that a higher *PIN* reflects higher information asymmetry in the stock market.

### Research design

To examine the effect of proxies for corporate transparency constructed from loan information on information asymmetry in the stock market, our baseline specification is as follows.

2 $$\begin{aligned} \begin{aligned} PIN_{i,t}&=\alpha +\beta _{1}\times Positive\_loan\_information_{i,t}/Loan\_default_{i,t}\\&\quad +\sum \beta _{i}\times Control_{i,t}+\varepsilon _{i,t} \end{aligned} \end{aligned}$$

where *i* is the firm index, *t* is the month index, and $PIN _{i,t}$ is the measure of information asymmetry in the stock market. A higher value of *PIN* indicates high information asymmetry in the stock market. $Control_{i,t}$ is a set of control variables, and $\varepsilon _{i,t}$ is the error term.

We include several controls,^5^ drawn from the prior literature (Sankaraguruswamy et al. 2013; Ma et al. 2019), related to information asymmetry: firm size (*Asset*), book-to-market (*BTM*), return on assets (*ROA*), market index (*Index 300*), type of market (*TM*), institutional shareholding ratio (*ISR*), analyst coverage (*Analyst*), trading volume (*Volume*), volatility (*Volatility*), and a dummy for highly leveraged small firms (*LAHL*). We control for firm size (*Asset*), because small firms have greater information asymmetry (Ma et al. 2019). We control for analyst coverage (*Analyst*) as a measure of information transparency^6^ because larger analyst coverage is negatively related to information asymmetry and analysts supply information to the market (Brennan and Subrahmanyam 1995; Roulstone 2003; Sankaraguruswamy et al. 2013). We include trading volume (*Volume*) and volatility (*Volatility *) as control variables, because prior research has documented a negative relationship between trading volume and *PIN* and a positive relationship between volatility and *PIN* (Sankaraguruswamy et al. 2013). We include a dummy for highly leveraged small firms (*LAHL*)^7^ because such firms are more likely to access syndicated loan markets than public ones (Maskara and Mullineaux 2011a, b). We also include firm and year $\times$ industry fixed effects. All continuous variables are winsorized with limits of 1% and 99%, and all estimations of the results are based on ordinary least squares, with errors clustered at the firm level.

We first examine the asymmetric effects of positive loan information (Hypothesis 1). The first set of loan information variables consists of *Loan *, *Loan size*, and *Tbank*. *Loan* is a dummy variable that equals 1 if a firm has at least one outstanding loan in a given month and equals 0 otherwise. *Loan size* is the total amount of the loan. *Tbank* is a dummy variable that equals 1 if the lending banks are joint-equity commercial banks and 0 otherwise. We expect the first set of positive loan information variables to have a negative $(\beta _{1})$ effect on the level of information asymmetry in the stock market.

We then examine the asymmetric information effects of loan defaults (Hypothesis 2). The second set of loan information comprises *OL*, *OL rate*, *OL Tbank*, and *OL Nbank*. *OL* is a dummy variable that equals 1 if a firm has at least one loan that fails to pay in a given month and equals 0 otherwise. *OL rate* is calculated as the overdue loan amounts divided by a firm’s total loan amounts. *OL Tbank* is a dummy variable that equals 1 if the loans fail to pay in a joint-equity commercial bank and equals 0 otherwise. *OL Nbank* is the total number of banks in which a firm has at least one overdue loan in a given month. We expect the second set of negative loan information variables to have a positive $(\beta _{1})$ effect on the level of information asymmetry in the stock market.

We also examine whether the asymmetric information effects of loan information vary with variability in the activity of the market condition (Hypothesis 3). We run baseline regressions and interact the loan information variables with the measure of market conditions. The regression equation is as follows:

3 $$\begin{aligned} \begin{aligned} PIN_{i,t}&=\alpha +\beta _{1}\times Positive\_loan\_information_{i,t}/Loan\_default_{i,t}\\&\quad +\beta _{2}\times Positive\_loan\_information_{i,t}/Loan\_default_{i,t}\times MC_{t}\\&\quad +\sum \beta _{i}\times Control_{i,t}+\varepsilon _{i,t} \end{aligned} \end{aligned}$$

where $MC_{t}$ is the market condition calculated as the market trading volume divided by the total number of shares outstanding in a given month. We expect that the interaction terms between loan and market turnover (*MC*) have a positive $(\beta _{2})$ effect on the information asymmetry in the stock market, and the interaction terms between overdue loan and market turnover have a negative $(\beta _{2})$ effect on the information asymmetry in the stock market.

### Data and descriptive statistics

Our data were obtained from several sources. Loan information is from a regulatory body that forms a dataset^8^ that includes monthly loan information from firms with credit lines greater than RMB 50 million that are extended by 17 major Chinese banks (the “big five state-owned banks” plus 12 joint-stock commercial banks) from January 2008 to June 2013. The Tinysoft database provides transaction data for all firms listed on the SSE that can be used to assess the direction of trade. From the Wind Information Inc. database, we obtain data on returns, trading, financial statements, and institutional shareholdings. We use the stock code to match each SSE-listed firm with its borrowing information from our loan information database. From all the above datasets, we exclude financial firms and firms with missing data. Our sample covers 1121 firms and 43,525 firm-month observations.

Table 1 provides a summary of the statistics for our sample. The average number of proxies for information asymmetry in the stock market *PIN* is 16%, which suggests that the Chinese stock market presents higher information asymmetry than the other markets. More than 65% of firms have outstanding loans with an average loan amount of RMB 492.33 million as corporate financing, and 46% of bank loans are borrowed from joint-equity commercial banks. On average, 3% of firms have at least one overdue loan, and 2% of bank loans fail to pay in a given month.

**Table 1:** Summary statistics

Variables	Mean	SD	Min	Median	Max	Obs.
*Dependent variable*						
PIN	0.16	0.09	0.00	0.14	0.54	43,525
*Positive loan information*						
Loan	0.65	0.48	0.00	1.00	1.00	43,525
Loan size (million)	492.33	1532.00	0.00	93.00	27,212.16	43,525
Tbank	0.46	0.50	0.00	0.00	1.00	43,525
*Loan default*						
OL	0.03	0.18	0.00	0.00	1.00	26,893
OL rate	0.02	0.12	0.00	0.00	1.00	26,893
OL Tbank	0.01	0.10	0.00	0.00	1.00	26,893
OL Nbank	0.03	0.18	0.00	0.00	1.00	26,893
*Others*						
Asset (billion)	53.65	135.05	0.13	20.83	4322.42	43,525
ROA	4.29	4.71	$-$ 6.10	3.15	22.55	43,525
BTM	0.31	0.20	0.01	0.27	1.02	43,525
Index 300	7.94	0.18	7.51	7.91	8.57	43,525
TM	0.50	0.50	0.00	0.00	1.00	43,525
ISR	0.31	0.22	0.00	0.28	0.83	43,525
Analyst	12.69	17.19	0.00	6.00	81	43,525
Volume (million)	1511.94	1703.61	148.65	955.56	10,679.32	43,525
Volatility	0.03	0.01	0.01	0.03	0.06	43,525
LAHL	0.03	0.17	0.00	0.00	1.00	43,525

This table provides summary statistics from January 2008 to June 2013. The full sample includes 43,525 firm-month observations, and the subsample, which is 26,893 firm-month observations, only includes firms with a bank loan. The definitions of the variables are given in Table 12 of Appendix 2, and all variables are winsorized with limits at 1% and 99%

## Empirical results

### Positive loan information and information asymmetry in the stock market

Table 2 presents the effects of outstanding loans on information asymmetry in the stock market. We regress *PIN* on loan information variables and a set of control variables. The three measures of corporate transparency are *Loan*, *Loan size*, and *Tbank*. The coefficients for *Loan*, *Loan size*, and *Tbank* are negative and statistically significant, as illustrated in Table 2.

Column 1 illustrates that the coefficient for the dummy variable *Loan* is negative and significant at the 10% level, consistent with our prediction that firms with outstanding loans have lower information asymmetry in the stock market. This decrease in *PIN* represents 28 basis points relative to firms without loans. As illustrated in Column 2, an increase of one standard deviation (1532 million) in loan size correlates to a 35.24 basis points reduction in *PIN*. Column 3 illustrates a coefficient of − 0.0028 for *Tbank*, with a *p* value less than 0.1, implying that joint-equity commercial banks provide more efficient monitoring than other banks. The level of information asymmetry in the stock market decreases with joint-equity commercial bank lending.

**Table 2:** Relationship between *PIN* and positive loan information

Variables	PIN		
	1	2	3
Intercept	0.2211***	0.3144***	0.3148***
	(8.45)	(11.42)	(11.44)
Loan	$-$ 0.0028*		
	($$-$$ 1.66)		
Loan size		$-$ 0.0023**	
		($$-$$ 2.22)	
Tbank			$-$ 0.0028*
			($$-$$ 1.70)
Asset	$-$ 0.0158*	$-$ 0.0084	$-$ 0.0132
	($-$ 1.88)	($-$ 0.92)	($-$ 1.48)
ROA	0.0002*	$-$ 0.0002	$-$ 0.0002
	(1.66)	($-$ 1.60)	($-$ 1.59)
BTM	0.0589***	0.0257***	0.0255***
	(14.10)	(4.95)	(4.91)
Index 300	$-$ 0.0124***	$-$ 0.0230***	$-$ 0.0230***
	($-$ 4.08)	($-$ 6.82)	($-$ 6.84)
TM	$-$ 0.0962*	0.1259***	0.1124***
	($-$ 1.89)	(5.75)	(5.15)
ISR	0.0430***	0.0108***	0.0110***
	(14.00)	(3.01)	(3.07)
Analyst	$-$ 0.0001**	$-$ 0.0001	$-$ 0.0001
	($-$ 2.55)	($-$ 1.38)	($-$ 1.40)
Volume	$-$ 0.0431***	$-$ 0.0592***	$-$ 0.0592***
	($-$ 15.43)	($-$ 19.21)	($-$ 19.20)
Volatility	0.0050	$-$ 0.0068	$-$ 0.0078
	(0.09)	($-$ 0.11)	($-$ 0.12)
LAHL	0.0018	0.0032	0.0033
	(0.32)	(0.64)	(0.65)
Year $\times$ industry-fixed effect	Yes	Yes	Yes
Firm-fixed effect	Yes	Yes	Yes
Adjusted $R^2$	0.0596	0.0792	0.0791
Obs.	43525	43525	43525

This table reports the ordinary least squares (OLS) results of the tests on the relationship between *PIN* and positive loan information. It represents the results of the regression: $PIN_{i,t}=\alpha +\beta _{1}\times Positive\_loan\_information_{i,t}+\sum \beta _{i}\times Control_{i,t}+\varepsilon _{i,t}$. *PIN* is the measure for information asymmetry in the stock market. The loan information variables are *Loan*, *Loan size*, and *Tbank*. The t-statistics reported are based on standard errors clustered by firm. Symbols *, **, and *** indicate significance at the 10%, 5%, and 1% levels, respectively. See Table 12 of Appendix 2 for variable definitions

The control variables are statistically significant. *PIN* is negatively related to firm size, market index, type of the market, analyst coverage, and trading volume, and is positively related to the book-to-market ratio and institutional shareholding ratio. Overall, the results provide evidence that positive loan information can reflect a lower level of information asymmetry in the stock market and that the asymmetric information effects are more pronounced if firms borrow more loans or borrow from joint-equity commercial banks. The results across all the specifications in Table 2 are consistent with H1.

### Negative loan information and information asymmetry in the stock market

Table 3 illustrates the univariate results based on two types of loans. We find that firms with overdue loans have a 100-basis point higher number in *PIN* than those without an overdue loan; this suggests that negative loan information may reflect a higher level of information asymmetry in the stock market.

**Table 3:** Portfolio analysis of PIN based on two types of loans

Variables	B1 (non-overdue loans)	B2 (overdue loans)	B1–B2
PIN	0.15	0.16	$-$ 0.01***
			(-3.36)

This table compares the *PIN* between the non-overdue loan samples and overdue loan samples. Symbols *, **, and *** indicate significance at the 10%, 5%, and 1% levels, respectively

For further analysis, we present the regressions *PIN* on overdue loans in Table 4, employing our second set of measures for corporate transparency constructed from negative loan information: *OL*, *OL rate*, *OL Tbank*, and *OL Nbank*. All regressions also contain a set of control variables, firm, and year $\times$ industry-fixed effects.

In Table 4, Columns 1 and 2 report a coefficient of 0.0116 for *OL*, with a *p* value less than 0.01, and a coefficient of 0.0325 for *OL rate*, with a *p* value less than 0.01. These results are economically significant. For example, a one standard deviation (0.12) increase in *OL rate* leads to a 39-basis point increment in the level of information asymmetry.

Columns 3 and 4 of Table 4 illustrate that the coefficients for *OL Tbank* and *OL Nbank* are positive and significant. Column 3 illustrates a coefficient of 0.0255 for *OL Tbank*, with a *p* value of less than 0.05. The asymmetric information effects of loan defaults are more pronounced when a firm borrows from a joint-equity commercial bank rather than a state-owned bank. Column 4 illustrates that an increase in *OL Nbank* represents a 116-basis point increment in *PIN*. The asymmetric information effects of loan defaults are particularly pronounced when loans are borrowed by multiple banks.

**Table 4:** Relationship between *PIN* and negative loan information

Variables	PIN			
	1	2	3	4
Intercept	0.3761***	0.3742***	0.3733***	0.3761***
	(10.51)	(10.47)	(10.41)	(10.51)
OL	0.0116***			
	(2.61)			
OL rate		0.0325***		
		(3.22)		
OL Tbank			0.0255**	
			(2.53)	
OL Nbank				0.0116***
				(2.61)
Asset	$-$ 0.0101	$-$ 0.0102	$-$ 0.0100	$-$ 0.0101
	($-$ 1.09)	($-$ 1.12)	($-$ 1.08)	($-$ 1.09)
ROA	$-$ 0.0001	$-$ 0.0001	$-$ 0.0001	$-$ 0.0001
	($-$ 0.54)	($-$ 0.47)	($-$ 0.43)	($-$ 0.54)
BTM	0.0218***	0.0225***	0.0226***	0.0218***
	(3.45)	(3.59)	(3.58)	(3.45)
Index 300	$-$ 0.0221***	$-$ 0.0219***	$-$ 0.0218***	$-$ 0.0221***
	($-$ 5.04)	($-$ 4.99)	($-$ 4.95)	($-$ 5.04)
TM	0.0277	0.0280	0.0274	0.0277
	(1.15)	(1.17)	(1.14)	(1.15)
ISR	0.0105**	0.0105**	0.0106**	0.0105**
	(2.20)	(2.20)	(2.20)	(2.20)
Analyst	$-$ 0.0001	$-$ 0.0001	$-$ 0.0001	$-$ 0.0001
	($-$ 1.15)	($-$ 1.17)	($-$ 1.17)	($-$ 1.15)
Volume	$-$ 0.0570***	$-$ 0.0570***	$-$ 0.0570***	$-$ 0.0570***
	($-$ 14.89)	($-$ 14.94)	($-$ 14.88)	($-$ 14.89)
Volatility	$-$ 0.0045	$-$ 0.0006	$-$ 0.0043	$-$ 0.0045
	($-$ 0.05)	($-$ 0.01)	($-$ 0.05)	($-$ 0.05)
LAHL	0.0009	$-$ 0.0007	0.0015	0.0009
	(0.10)	($-$ 0.08)	(0.17)	(0.10)
Year $\times$ industry-fixed effect	Yes	Yes	Yes	Yes
Firm-fixed effect	Yes	Yes	Yes	Yes
Adjusted $R^2$	0.0683	0.0684	0.0684	0.0683
Obs.	26893	26893	26893	26893

This table reports the OLS results of the tests on the relationships between *PIN* and overdue loans. It represents the results of the regression: $PIN_{i,t}=\alpha +\beta _{1}\times Loan\_default_{i,t}+\sum \beta _{i}\times Control_{i,t}+\varepsilon _{i,t}$. *PIN* is the measure for information asymmetry in the stock market. The variables for bad news in the loan market in this table are *OL*, *OL rate*, *OL Tbank*, and *OL Nbank*. The t-statistics reported are based on standard errors clustered by firm. Symbols *, **, and *** indicate significance at the 10%, 5%, and 1% levels, respectively. See Table 12 of Appendix 2 for variable definitions

Overall, our evidence suggests that a firm’s opacity, caused by negative loan information, increases information asymmetry in the stock market. These findings are consistent with our expectations and H2.

By comparing Tables 2 and 4, we see that the asymmetric information effects caused by negative loan information are much larger than those caused by positive loan information. These findings indicate that negative loan information contains more innovations in stock market information than positive loan information. As corporate public disclosure contains more good news than bad news, negative loan information can serve as a good proxy for a lower level of corporate transparency.

### Market conditions

To examine the asymmetric information effects under different market conditions, we use market turnover as a proxy for market conditions, calculated as the market trading volume divided by the total number of shares outstanding in a given month. A higher market turnover indicates more active market conditions. As illustrated in Panel A of Table 5, the coefficients for the interaction terms between loans and market turnover are all positive, suggesting that the asymmetric information effects are lower under active market conditions.

**Table 5:** Market conditions analysis

Variables	PIN			
	1	2		3
*Panel A: Loans on information asymmetry in the stock market in different market conditions*				
Loan	$-$ 0.0070*			
	($$-$$ 2.05)			
Loan$\times$MC	0.0051			
	(1.06)			
Loan size		$-$ 0.0028**		
		($$-$$ 2.37)		
Loan size $\times$ MC		0.0011		
		(0.92)		
Tbank				$-$ 0.0099***
				($$-$$ 3.10)
Tbank $\times$ MC				0.0123***
				(2.75)
Controls	Yes	Yes		Yes
Year $\times$ industry-fixed effect	Yes	Yes		Yes
Firm-fixed effect	Yes	Yes		Yes
Adjusted $R^2$	0.0597	0.0799		0.0800
Obs.	43525	43525		43525

Variables	PIN			
	1	2	3	4
*Panel B: Overdue loans on information asymmetry in the stock market in different market conditions*				
OL	0.0255***			
	(2.68)			
OL $\times$ MC	$-$ 0.0233*			
	($$-$$ 1.90)			
OL rate		0.0523***		
		(3.04)		
OL rate $\times$ MC		$-$ 0.0341*		
		($$-$$ 1.94)		
OL Tbank			0.0518**	
			(2.09)	
OL Tbank $\times$ MC			$-$ 0.0430	
			($$-$$ 1.54)	
OL Nbank				0.0255***
				(2.68)
OL Nbank $\times$ MC				$-$ 0.0233*
				($$-$$ 1.90)
Controls	Yes	Yes	Yes	Yes
Year $\times$ industry-fixed effect	Yes	Yes	Yes	Yes
Firm-fixed effect	Yes	Yes	Yes	Yes
Adjusted $R^2$	0.0663	0.0663	0.0663	0.0663
Obs.	26893	26893	26893	26893

This table reports the OLS results of the tests on the relationships between *PIN* and bank loan information in different market conditions. It represents the results of the regression: $PIN_{i,t}=\alpha +\beta _{1}\times Positive\_loan\_information_{i,t}/Loan\_default_{i,t}+\beta _{2}\times Positive\_loan\_information_{i,t}/Loan\_default_{i,t}\times MC_{i,t}+\sum \beta _{i}\times Control_{i,t}+\varepsilon _{i,t}$, with *PIN* as a measure for information asymmetry in the stock market and MC as a variable representing market turnover. Panel A provides the regressions of loans on information asymmetry in the stock market in different market conditions. Panel B provides the regressions of overdue loans on information asymmetry in the stock market in different market conditions. The t-statistics reported are based on standard errors clustered by firm. Symbols *, **, and *** indicate significance at the 10%, 5%, and 1% levels, respectively. See Table 12 of Appendix 2 for variable definitions

In Panel B of Table 5, the coefficients for the interaction terms between overdue loans and market turnover are negative and significant at the 10% level; this indicates that the asymmetric information effects of loan defaults will be less pronounced under active market conditions.

Moreover, the coefficients for the interaction terms between overdue loan information and market turnover are more significant than those for positive information. This is consistent with the findings presented in Tables 2 and 4. Once again, this result indicates that negative loan information conveys more effective identification for corporate transparency; that is, firms are more willing to report good news than bad news. Bank loan information provides more quality information than voluntary disclosure, especially negative loan information.

## Additional analyses

### Selection bias

One potential endogeneity issue in our analyses is that whether a firm can obtain a bank loan correlates with its fundamentals and market conditions, which can also affect its information transparency. This possibility creates a misrepresentation in our sample since firms with good fundamentals are also more likely to obtain loans.

However, we add firm fixed effects to control the time-invariant heterogeneity of firms in our baseline model. To further control for this potential sample selection bias, we apply the Heckman two-stage selection model.

**Table 6:** Heckman’s two-stage model results

Variables	The second stage results					
	PIN					
	1	2	3	4	5	6
Intercept	0.3965***	0.4035***	0.3938***	0.3930***	0.3913***	0.3925***
	(5.74)	(5.84)	(5.70)	(5.69)	(5.67)	(5.68)
Loan size	$-$ 0.0028***					
	($$-$$ 3.07)					
Tbank		$-$ 0.0047***				
		($$-$$ 2.64)				
OL			0.0084**			
			(2.03)			
OL rate				0.0261***		
				(2.63)		
OL Tbank					0.0209***	
					(2.70)	
OL Nbank						0.0048*
						(1.96)
Lambda	$-$ 0.0056	0.0054	0.0055	$-$ 0.0042	$-$ 0.0042	$-$ 0.0041
	($-$ 1.11)	(1.36)	(1.38)	($-$ 0.84)	($-$ 0.83)	($-$ 0.81)
Controls	Yes	Yes	Yes	Yes	Yes	Yes
Year $\times$ industry-fixed effect	Yes	Yes	Yes	Yes	Yes	Yes
Firm-fixed effect	Yes	Yes	Yes	Yes	Yes	Yes
Adjusted $R^2$	0.0805	0.0805	0.0804	0.0758	0.0758	0.0757
Obs.	27025	27025	27025	27025	27025	27025

This table reports the results of the second stage of the Heckman (1979) two-step procedure that considers the potential selection bias. The dependent variable in the first-stage probit regression is a dummy variable that equals 1 if a firm has at least one outstanding loan in a given month and equals 0 otherwise. In the second stage, we estimate Eq. (2) including an additional control variable equal to the inverse Mills ratio obtained from the first stage. The t-statistics reported are based on standard errors clustered by firm. Symbols *, **, and *** indicate significance at the 10%, 5%, and 1% levels, respectively

Table 6 reports the results of the second stage of Heckman (1979) ’s two-step procedure. The dependent variable in the first-stage probit regression^9^ is a dummy variable, *Loan*, which equals 1 if a firm has at least one outstanding loan in a given month, and 0 otherwise. In the second stage, we estimate Eq. (2), including an additional control variable equal to Heckman’s lambda obtained in the first stage. None of the coefficients for Heckman’s lambda are significant, which means that the selection bias issue does not exist. The results are identical to the regression findings in Tables 2 and 4 in terms of coefficient signs and significance. These results indicate that a sample selection bias did not drive our former findings.

### Volume-synchronized probability of informed trading and bid-ask spread

We use *VPIN* (Volume-Synchronized Probability of Informed Trading) (Easley et al. 2012) and *Bid-Ask Spread* (Lee et al. 1993; Madhavan et al. 1997) as measures of information asymmetry in the stock market to ensure the robustness of our findings.

Easley et al. (2012) improves the algorithm of *PIN* and proposes *VPIN*. *VPIN* is defined as the absolute value of the difference between the sell and buy trades divided by total trades. As the Chinese stock market can provide transaction information, we do not need to adopt a method to split the volume Easley et al. (2012).

*Bid-Ask Spread*, which measures market liquidity, is calculated as the difference between the bid price and the ask price. The smaller the spread in stock trading, the higher the liquidity of stock markets. This represents a decrease in the extent of information asymmetry.

We use *VPIN* and *Bid-Ask Spread* to replace *PIN* as the dependent variable to re-estimate the information asymmetry effects; the results are illustrated in Table 7. Consistent with the results in Tables 2 and 4, the coefficients for *Loan size* are significantly negative in Columns 1 and 3, and the coefficients for *OL rate* are significantly positive in Columns 2 and 4. The coefficient for *OL rate* is significantly larger than the absolute value of the coefficient for *Loan size*. These findings provide consistent evidence that information opacity in the loan market increases information asymmetry in the stock market.

**Table 7:** Relationship between *VPIN* or *Bid-Ask Spread* and loan information

Variables	VPIN		Bid-Ask Spread	
	1	2	3	4
Loan size	$-$ 0.0017**		$-$ 0.0067*	
	($-$ 2.47)		($-$ 1.74)	
OL rate		0.0065***		0.0461***
		(2.94)		(4.53)
Controls	Yes	Yes	Yes	Yes
Year $\times$ industry-fixed effect	Yes	Yes	Yes	Yes
Firm-fixed effect	Yes	Yes	Yes	Yes
Adjusted $R^2$	0.4782	0.4628	0.6470	0.6689
Obs.	43,525	26,893	43,525	26,893

This table reports the OLS results of the tests on the relationships between *VPIN* or *Bid-Ask Spread* and loan information. It represents the results of the regression: $VPIN_{i,t}/Bid-Ask Spread_{i,t}=\alpha +\beta _{1}\times Positive\_loan\_information_{i,t}/Loan\_default_{i,t}+\sum \beta _{i}\times Control_{i,t}+\varepsilon _{i,t}$, with *VPIN* defined as the absolute value of the difference between sell trades and buy trades divided by total trades. *Bid-Ask Spread* is calculated as the difference between bid price and ask price, to measure market liquidity. The control variables in previous tables are included in the regressions. The t-statistics reported are based on standard errors clustered by firm. Symbols *, **, and *** indicate significance at the 10%, 5%, and 1% levels, respectively

### New loan information

We use new loan information as an additional measure of corporate transparency. *New Loan* is a dummy variable that indicates that a firm obtains at least one new loan in a given month, which means lenders can gather new information about borrowers during this loan transaction to ensure the timely repayment of *loans* and interest that are their claims on the borrowers’ future cash flow and assets.

Panel A of Table 8 reports the new positive loan information. The coefficients for *New Loan* and *New Loan * are both significant and negative. These results demonstrate that firms with new loans have lower information asymmetry in the stock market, consistent with Table 2’s findings.

**Table 8:** Relationship between PIN and new loan information

Variables	PIN			
	1	2		3
*Panel A: Relation between PIN and new loans*				
Intercept	0.4131***	0.3141***		0.3136***
	(14.71)	(11.38)		(11.36)
New loan	$-$ 0.0031**			
	($$-$$ 2.26)			
New loan size		$-$ 0.0050*		
		($$-$$ 1.75)		
New Tbank				$-$ 0.0013
				($$-$$ 0.85)
Controls	Yes	Yes		Yes
Year $\times$ industry-fixed effect	Yes	Yes		Yes
Firm-fixed effect	Yes	Yes		Yes
Adjusted $R^2$	0.0600	0.0791		0.0791
Obs.	43,525	43,525		43,525

Variables	PIN			
	1	2	3	4
*Panel B: Relation between PIN and new overdue loans*				
Loan information (LI)	OL	OL rate	OL Tbank	OL Nbank
Intercept	0.4200***	0.4171***	0.4181***	0.4197***
	(10.64)	(10.58)	(10.61)	(10.62)
New LI	0.0019	0.0287	0.0168	0.0022
	(0.33)	(1.03)	(1.16)	(0.48)
New LI t-1	0.0032	0.0420	0.0221	0.0041
	(0.44)	(1.63)	(1.16)	(0.73)
New LI t-2	$-$ 0.0064	$-$ 0.0212	$-$ 0.0081	$-$ 0.0082**
	($-$ 1.30)	($-$ 0.98)	($-$ 0.93)	($-$ 2.00)
New LI t-3	$-$ 0.0132***	$-$ 0.0129	$-$ 0.0219***	$-$ 0.0094**
	($-$ 2.79)	($-$ 0.67)	($-$ 2.64)	($-$ 2.08)
Controls	Yes	Yes	Yes	Yes
Year $\times$ industry-fixed effect	Yes	Yes	Yes	Yes
Firm-fixed effect	Yes	Yes	Yes	Yes
Adjusted $R^2$	0.0687	0.0686	0.0687	0.0687
Obs.	26,022	26,022	26,022	26,022

This table reports the OLS results of the tests on the relationships between *PIN* and new loan information. It represents the results of the regression: $PIN_{i,t}=\alpha +\beta _{1}\times Positive\_loan\_information_{i,t}/Loan\_default_{i,t}+\sum \beta _{i}\times Control_{i,t}+\varepsilon _{i,t}$, where *PIN* is the measure for information asymmetry in the stock market. Variables of new loans in Panel A are *New Loan*, *New Loan Size*, and *New Tbank*. Variables of new overdue loans in Panel B are *New OL*, *New OL rate*, *New OL Tbank*, and *New OL Nbank*. The control variables in previous tables are included in the regressions, and the t-statistics reported are based on standard errors clustered by firm. Symbols *, **, and *** indicate significance at the 10%, 5%, and 1% levels, respectively

Panel B of Table 8 presents the results for the new negative loan information. Variable *New OL* indicates that a firm fails to pay at least one new overdue loan. The coefficients for *New OL* and *New OL Tbank* are significant and negative only for the 3-month lagging indicators. The coefficients for *New OL Nbank* are significant and negative for the 2- and 3-month lag indicators. The reaction to negative loan information in the equity market lags behind that to positive loan information.

The asymmetric information effects caused by new loans and new overdue loans are weaker than the effects caused by outstanding loans and overdue loans. Further, the effects of new overdue loans even lag by 3 months. As not all loan information is publicly disclosed, it takes time for loan information to transfer from the loan market to the stock market, and good private news will generally be released faster than bad private news.

### Non-performing loan

Next, we use NPLs to replace overdue loans to measure information opacity. Among the five classifications of loans—normal, concerned, sublevel, doubted, and loss—the last three levels are regarded as non-performing loans according to the People’s Bank of China’s loan classification guidelines. In contrast to loan overdue, marking a loan as non-performing depends on a loan officer’s subjective judgment, and the decision could be influenced by human factors.^10^ Therefore, this negative loan information does not seem to be easy to share efficiently with stock markets.

Table 9 reports the NPL results from our data. The coefficient for *NPL rate* is significant and positive, and the coefficients for *NPL*, *NPL Tbank*, and *NPL Nbank* are positive. These results demonstrate that the existence of non-performing loans reflects a higher level of information asymmetry in the stock market and is consistent with the findings in Table 4.

**Table 9:** Relationship between *PIN* and *NPL*

Variables	PIN			
	1	2	3	4
NPL	0.0084			
	(1.12)			
NPL rate		0.0199**		
		(2.03)		
NPL Tbank			0.0057	
			(0.66)	
NPL Nbank				0.0034
				(1.37)
Controls	Yes	Yes	Yes	Yes
Year $\times$ industry-fixed effect	Yes	Yes	Yes	Yes
Firm-fixed effect	Yes	Yes	Yes	Yes
Adjusted $R^2$	0.0681	0.0682	0.0681	0.0681
Obs.	26,893	26,893	26,893	26,893

This table reports the OLS results of the tests on the relationships between *PIN* and non-performing loans. It represents the results of the regression: $PIN_{i,t}=\alpha +\beta _{1}\times Loan\_default_{i,t}+\sum \beta _{i}\times \times Control_{i,t}+\varepsilon _{i,t}$, where *PIN* is a measure for information asymmetry in the stock market. In this table, variables of bad news in the loan market are *NPL*, *NPL rate*, *NPL Tbank*, and *NPL Nbank*. The control variables in previous tables are included in the regressions, and the t-statistics reported are based on standard errors clustered by firm. Symbols *, **, and *** indicate significance at the 10%, 5%, and 1% levels, respectively

### Regulation fair disclosure

Privileged information about borrowers is only obtained by their lenders and is not publicly available. Although national-level laws and regulations require listed companies to disclose such information, lenders will always obtain private information earlier than public investors. In China, the “Administrative Measures for the Disclosure of Information of Listed Companies” and “Compilation Rules for Information Disclosure by Companies Offering Securities to the Public” require the disclosure of loan default; however, the details and timing for the disclosure of loan default are not clearly defined. The borrower is not required to disclose information to the public. Further, default information about borrowers may not be disclosed or may be disclosed at a later time.

In Table 10, we remove observations that contain publicly announced overdue loan information and re-estimate the relationship between *PIN* and overdue loans. These results are consistent with those presented in Table 4. The findings suggest that loan information without disclosure of overdue loans reflects information asymmetry in the stock market.

**Table 10:** Relationship between *PIN* and overdue loans excluding disclosure

Variables	PIN			
	1	2	3	4
OL	0.0115**			
	(2.55)			
OL rate		0.0336***		
		(3.26)		
OL Tbank			0.0269**	
			(2.53)	
OL Nbank				0.0116**
				(2.55)
Controls	Yes	Yes	Yes	Yes
Year $\times$ industry-fixed effect	Yes	Yes	Yes	Yes
Firm-fixed effect	Yes	Yes	Yes	Yes
Adjusted $R^2$	0.0690	0.0691	0.0691	0.0690
Obs.	26,845	26,845	26,845	26,845

This table reports the OLS results of the tests on the relationships between *PIN* and overdue loans in the subsample where news disclosure is excluded. It represents the results of the regression: $PIN_{i,t}=\alpha +\beta _{1}\times Loan\_default_{i,t}+\sum \beta _{i}\times Control_{i,t}+\varepsilon _{i,t}$, where *PIN* is a measure for information asymmetry in the stock market. Variables for bad news in the loan market in this table are *OL*, *OL rate*, *OL Tbank*, and *OL Nbank*. The control variables in previous tables are included in the regressions, and the t-statistics reported are based on standard errors clustered by firm. Symbols *, **, and *** indicate significance at the 10%, 5%, and 1% levels, respectively

## Conclusion

This study uses a novel dataset of stock transactions and loan information of all publicly listed firms on the SSE from January 2008 to June 2013 to analyze the effect of corporate transparency measured by bank loan information on information asymmetry in the stock market. Our results indicate that positive (negative) loan information reflects a lower (higher) level of information asymmetry in the stock market. The main findings of this study are summarized as follows.

First, we provide evidence that firms with outstanding loans have lower levels of information asymmetry in the stock market. Our analysis demonstrates that proxies for corporate transparency constructed from loan information reflect information asymmetry in the stock market. Furthermore, this effect is more pronounced if the firm borrows a larger loan or borrows from a joint-equity commercial bank.

Second, we prove that the information opacity measured by negative loan information (loan defaults) can reflect a higher level of information asymmetry in the stock market. The asymmetric information effects caused by loan defaults are more pronounced when firms borrow from joint-equity commercial banks and multiple banks. These findings suggest that financially distressed firms (firms with defaulted loans) tend to hide negative information, which increases both the information asymmetry between borrowers and lenders and the level of information asymmetry in the stock market.

Third, we consider market conditions and find that the asymmetric information effects of loan defaults are less pronounced under active market conditions.

Finally, our results are robust to different samples and specifications and a series of endogeneity and robustness tests. Our findings remain largely the same when we consider potential issues caused by sample selection bias, use different measures for information asymmetry in the stock market and loan information, and conduct an analysis based on a subsample that does not include observations with public loan information announcements.

In conclusion, our empirical results shed light on the adequacy of using loan information to construct corporate transparency measures. While prior studies generally focus on corporate disclosure, we provide evidence that loan information captures another aspect of corporate transparency, because banks have preferred access to firms’ private information and can collect both positive and negative information. Our findings examine the relationship between bank loan information and information asymmetry in the stock market, complementing the findings in the extant literature. We provide new evidence that firms with outstanding loans have a lower level of information asymmetry in the stock market and that loan defaults can reflect a higher level of information asymmetry in the stock market. These findings are consistent with the literature that financially distressed firms are more informationally opaque and have a higher level of information asymmetry in the stock market. Future studies could be undertaken to analyze the asymmetric information effects of loan information under extreme situations, such as the 2008 financial crisis and COVID-19 pandemic, or considering economic policy uncertainty.

## Data Availability

The data supporting the findings of this study are available upon request from the corresponding author. The data are not publicly available because of privacy or ethical restrictions.

## Appendices

### Appendix 1: Bank-related financial institutions

Table 11 presents bank-related financial institutions: the relationships, funds, and securities firms

**Table 11:** Bank-related financial institutions

Banks	Relationship	Funds	Securities firms
China CITIC Bank	Same group	Xincheng Fund Management Co., Ltd.	CITIC Securities Co., Ltd.
		China Asset Management Co., Ltd.	
Shanghai Pudong development bank	Holding	AXA-SPDB Investment Managers Co., Ltd.	
China construction bank	Holding	CCB Principal Asset Management Co., Ltd.	
China’s Industrial Bank	Holding	CIB Fund Management Co., Ltd.	
Bank of communications	Holding	Bank of Communications Schroder Fund Management Co., Ltd.	
China Bohai BANK	Same group	ManulifeE TEDA Fund Management Co., Ltd.	
Industrial and commercial bank	Holding	Icbc Credit Suisse Asset Management Co., Ltd.	
Bank of China	Holding	Bank Of China Investment Management Co., Ltd.	
China Minsheng Bank	Holding	Minsheng Royal Fund Management Co., Ltd.	
Guangdong Development Bank	Same group	GF Fund Management Co., Ltd.	GF Securities Co., Ltd.
China Merchants Bank	Holding	China Merchants Fund Management Co.,Ltd.	
China Zhe Shang Bank	Same group	ZheShang Fund Management Co.,Ltd	Zheshang Securities Co., Ltd.
Agricultural Bank of China	Holding	ABC-CA Fund Management Co., Ltd	
China Ping An Bank	Same group	Ping An Fund Management Co., Ltd	Ping An Securities Co., Ltd.
China Everbright Bank	Same group	Everbright Pramerica Fund Management Co.,Ltd.	Everbright Securities Co., Ltd.
		Da Cheng Fund Management Co., Ltd.	

### Appendix 2: Definition of variables

Table 12 provides a detailed definition of the main variables used in the analysis.

**Table 12:** Definition of variables

Variables	Definition
PIN	Probability of information-based trades Easley et al. (2002).
Loan	A dummy variable that equals 1 if a firm has at least one outstanding loan in a given month and equals 0 otherwise
Loan size	Total amount of the loan (in million RMB)
Tbank	A dummy variable that equals 1 if the lending bank is a joint-equity commercial bank and equals 0 otherwise
OL	A dummy variable that equals 1 if a firm has at least one loan that fails to pay in a given month and equals 0 otherwise
OL rate	OL rate is calculated as the overdue loans’ amounts divided by a firm’s total loan amounts
OL Tbank	A dummy variable that equals 1 if the overdue loan is with a commercial bank and equals 0 otherwise
OL Nbank	OL Nbank is the total number of banks, in which that firm has at least one overdue loan in a given month
New loan	A dummy variable that equals 1 if a firm gets at least one new outstanding loan in a given month and equals 0 otherwise
New loan size	Total amount of the new loans (in million RMB)
New Tbank	A dummy variable that equals 1 if the lending bank that offers the new loans is a commercial bank and equals 0 otherwise
NPL	A dummy variable that equals 1 if a firm has at least one non-performing loan in a given month and equals 0 otherwise
NPL rate	NPL rate is calculated as the non-performing loan’s amount divided by a firm’s total loan amounts
NPL Tbank	A dummy variable that equals 1 if the NPL loan is in a commercial bank and equals 0 otherwise
NPL Nbank	NPL Nbank is the total number of banks in which that firm has at least one NPL loan in a given month
Asset	The book value of the firm’s total assets. (in billion RMB)
ROA	Return on asset (%)
BTM	Book to market ratio is calculated as the firm’s book value divided by the firm’s market value
Index 300	Index 300 is a capitalization-weighted stock market index designed to replicate the performance of 300 stocks traded in the Shanghai and Shenzhen stock exchanges
TM	A dummy variable that equals 1 if the firm is listed in Shenzhen main board and equals 0 otherwise
ISR	Institutional investors holding ratio is calculated as the shares held by institutional investors divided by all shares of the firm
Analyst	Analyst is the number of analysts covering the firm, calculated as the number of analysts with earnings forecasts for the current fiscal year
Volume	Volume is the trading dollar volume in a given month (in million RMB)
Volatility	Volatility is standard deviation of the return in a given month
LAHL	A dummy variable that equals 1 if a firm is in the lowest quartile based on total assets and also in the highest quartile based on leverage, and equals 0 otherwise
INTAN	Intangible asset ratio is calculated as intangible assets to total assets
MC	MC is market conditions, calculated as the market trading volume divided by the total number of shares outstanding in a given month
Leverage	Leverage is the ratio of long-term debt to total assets
Z-Score	Altman’s (1968) Z-score
TAN	Tangible asset ratio is calculated as tangible assets to total assets

### Appendix 3: Robust test using an alternative proxy for corporate transparency

Table 13 illustrates the relationship between *PIN* and loan information after adding the intangible asset ratio as a proxy for corporate transparency.

**Table 13:** Relationship between PIN and loan information after adding the intangible asset ratio

Variables	PIN			
	1	2		3
*Panel A: Relation between PIN and corporate transparency by adding intangible asset ratio*				
Intercept	0.3125***	0.3131***		0.3137***
	(11.33)	(11.36)		(11.38)
Loan	− 0.0029*			
	(− 1.65)			
Loan size		− 0.0023**		
		(− 2.26)		
Tbank				− 0.0031*
				(− 1.86)
INTAN	0.0306	0.0320		0.0317
	(1.28)	(1.33)		(1.32)
Year $\times$ industry-fixed effect	Yes	Yes		Yes
Firm-fixed effect	Yes	Yes		Yes
Adjusted $R^2$	0.0800	0.0801		0.0801
Obs.	43,088	43,088		43,088

Variables	PIN			
	1	2	3	4
*Panel B: Relation between PIN and overdue loans by adding intangible asset ratio*				
Intercept	0.3820***	0.3802***	0.3790***	0.3820***
	(10.61)	(10.56)	(10.50)	(10.61)
OL	0.0116***			
	(2.59)			
OL rate		0.0318***		
		(3.12)		
OL Tbank			0.0254**	
			(2.51)	
OL Nbank				0.0116***
				(2.59)
INTAN	0.0452	0.0426	0.0473	0.0452
	(1.28)	(1.20)	(1.35)	(1.28)
Year $\times$ industry-fixed effect	Yes	Yes	Yes	Yes
Firm-fixed effect	Yes	Yes	Yes	Yes
Adjusted $R^2$	0.0695	0.0696	0.0696	0.0695
Obs.	26,625	26,625	26,625	26,625

Symbols *, **, and *** indicate significance at the 10%, 5%, and 1% levels, respectively

### Appendix 4: First stage of the Heckman two-step procedure

Table 14 presents the results of the first stage of the Heckman two-step procedure.

**Table 14:** First stage of the Heckman two-step procedure

Variables	Probit regression
	loan
Intercept	$-$ 0.5658***
	($$-$$ 3.79)
Asset	0.0446
	(0.46)
ROA	0.0132***
	(7.98)
BTM	1.2497***
	(25.80)
Leverage	2.4251***
	(51.71)
Z-Score	0.4759***
	(3.34)
TAN	$-$ 0.0313***
	(− 24.51)
Year-fixed effect	Yes
Industry-fixed effect	Yes
Pseudo $R^2$	0.2022
Obs.	40848

This table reports the results of the first stage of the Heckman two-step procedure estimated by a Probit regression. The independent variables are *Asset*, *ROA*, *BTM*, *Leverage*, *Z-Score*, *TAN* (Graham et al. 2008). Symbols *, **, and *** indicate significance at the 10%, 5%, and 1% levels, respectively

## Footnotes

1 In China, indirect financing makes up more than 80% of company financing. Compared with other financial institutions, banks have the most comprehensive and accurate micro-level enterprise information.

2 Table 11 of Appendix 1 illustrates the bank-related financial institutions.

3 Firms with outstanding loans and with a new loan in a given month are treated as positive loan information, while firms with overdue/non-performing loans in a given month are considered to be negative loan information. This expression is used across the whole paper.

4 The asymmetric information effects refer to the effect of bank loan information on information asymmetry in the stock market. This expression is used across the whole paper.

5 Definitions of variables are given in Table 12 of Appendix 2.

6 We use intangible asset ratio as a measure for information transparency in Table 13 of Appendix 3.

7 We follow Maskara and Mullineaux (2011b) and construct *LAHL* as a dummy variable. We also use different cutoffs (median vs. quantile) or sorting methods (dependent sorting vs. independent sorting) to construct *LAHL*, and all results remain the same.

8 Loan information dataset in this paper is from the same source as Cong et al. (2019).

9 Table 14 of Appendix 4 illustrates the results of the first-stage Heckman two-step procedure.

10 The correlation between *OL* and *NPL* is 88.49%.
